# A new marsh beetle from mid-Cretaceous amber of northern Myanmar (Coleoptera: Scirtidae)

**DOI:** 10.1038/s41598-022-16822-y

**Published:** 2022-08-04

**Authors:** Yan-Da Li, Rafał Ruta, Erik Tihelka, Zhen-Hua Liu, Di-Ying Huang, Chen-Yang Cai

**Affiliations:** 1 State Key Laboratory of Palaeobiology and Stratigraphy, Nanjing Institute of Geology and Palaeontology, and Centre for Excellence in Life and Palaeoenvironment, Chinese Academy of Sciences, Nanjing, 210008 People’s Republic of China; 2 Department of Biodiversity and Evolutionary Taxonomy, University of Wrocław, Przybyszewskiego 65, 51-148 Wrocław, Poland; 3 School of Earth Sciences, Life Sciences Building, University of Bristol, Tyndall Avenue, Bristol, BS8 1TQ UK; 4 State Key Laboratory of Biocontrol, School of Life Sciences, Sun Yat-Sen University, Guangzhou, 510275 China; 5 Australian National Insect Collection, CSIRO National Research Collections Australia, GPO Box 1700, Canberra, ACT 2601 Australia

**Keywords:** Palaeontology, Phylogenetics, Taxonomy, Entomology

## Abstract

As one of the earliest-diverging lineage of the megadiverse beetle suborder Polyphaga, marsh beetles (Scirtidae) are crucial for reconstructing the ancestor of all polyphagan beetles and the ecomorphological underpinnings of their remarkable evolutionary success. The phylogeny of marsh beetles has nonetheless remained challenging to infer, not least because of their fragmentary Mesozoic fossil record. Here we describe a new scirtid beetle genus and species, *Varcalium lawrencei* gen. et sp. nov*.*, preserving internal tissue, from Albian–Cenomanian Kachin amber (*ca* 99 Ma), representing the second member of this family known from the deposit. Based on a formal morphological phylogenetic analysis, *Varcalium* is recovered within the crown-group of Scirtinae, forming a clade with other genera that possess subocular carinae. The finding suggests that the crown-group of Scirtinae has already diversified by the mid-Cretaceous.

## Introduction

The marsh beetles (Scirtidae) are a globally distributed group of insufficiently known beetles associated with aquatic habitats. Marsh beetles have been traditionally placed in the superfamily Scirtoidea and regarded as one of the earliest-diverging groups within the megadiverse suborder Polyphaga^1^. Recent molecular phylogenetic analyses suggest that the classical concept of Scirtoidea (sensu Bouchard et al.^2^) is not monophyletic and instead the families Scirtidae and Decliniidae form the earliest-diverging clade within Polyphaga^3–5^. Reflecting these findings and morphological evidence, the concept of Scirtoidea is to be revised to include only Scirtidae, Decliniidae, and poorly-known extinct groups^5^. In light of these findings, Scirtidae represents a key taxon for reconstructing the last common ancestor of the suborder Polyphaga and hence the origins of its remarkable diversification.

While Decliniidae is a small family with one genus and two described species known from the Northeast Asia only^6–8^, Scirtidae is cosmopolitan, with around 100 genera and about 1900 described species^9^. The larvae of Scirtidae are usually aquatic detritus feeders, with adults often short-lived. The internal relationships of Scirtidae are poorly understood. Although recently years have witnessed a growing number of studies on various scirtid groups (e.g.,^10–28^), generic misplacements within the family seem to remain prevalent. The molecular phylogenetic studies of Scirtidae conducted to date, though with a focus on Australian taxa only, have nevertheless demonstrated that many currently recognised genera are not monophyletic and that scirtid diversity at the generic level is severely underestimated^29–32^.

Most scirtid fossils are known from Eocene Baltic amber (as listed by Alekseev^33^). Many of them are well preserved with exposed genitalia and most are placed in extant genera (e.g., *Contacyphon* Gozis, *Elodes* Latreille, *Microcara* Thomson^34,35^), but several new genera were also described^36^. Additional scirtids were reported (though some of them were not officially named) from Lower Cretaceous Lebanese amber, Lower Cretaceous deposits in Koonwarra (Australia), and Eocene Fushun and Oise ambers^37–39^. Though the mid-Cretaceous Kachin amber from northern Myanmar preserved a diverse fauna of insects and other terrestrial arthropods^40–42^, scirtids appear to be relatively uncommon in Kachin amber. The only previously described scirtid from Kachin amber is *Mesernobius anawrahtai* Engel, which was poorly illustrated and superficially described, and was originally attributed to the family Ptinidae^43^. Peris et al.^44^ transferred *Mesernobius* Engel to Scirtidae mainly based on the deflexed head, short and broad prothorax, and the bilobed fourth tarsomere. However, the ventral structures of head and thorax of *Mesernobius* remain unclear and will be crucial for determining its systematic position within Scirtidae.

Recently we have found several scirtids in our collection of Kachin amber. Here, we report a new genus of crown-group Scirtidae from Kachin amber, which, together with other unpublished scirtids, suggests that the family was more diverse in the Kachin amber rainforest than previously thought.

## Systematic Palaeontology

Order Coleoptera Linnaeus, 1758.

Suborder Polyphaga Emery, 1886.

Superfamily Scirtoidea Fleming, 1821.

Family Scirtidae Fleming, 1821.

*Varcalium* Li, Ruta, Tihelka & Cai gen. nov.

**Type species.** *Varcalium lawrencei* sp. nov., here designated.

**Etymology.** The generic name is an anagram of *Calvarium* Pic, a genus in Scirtidae. The name is masculine in gender.

**Diagnosis.** Antennae serrate. Subocular carina present, smoothly connecting supraantennal ridge with subgenal ridge. Subgenal ridge without buttonhole configuration (sensu Zwick^22^), situated close to eye. Clypeus with deep, rectangular emargination. Anterior mesoventral margin without notch for prosternal process. Hind femora not distinctly thickened.

*Varcalium lawrencei* Li, Ruta, Tihelka & Cai sp. nov.

(Figs. 1, 2, 3).

**Figure 1:**
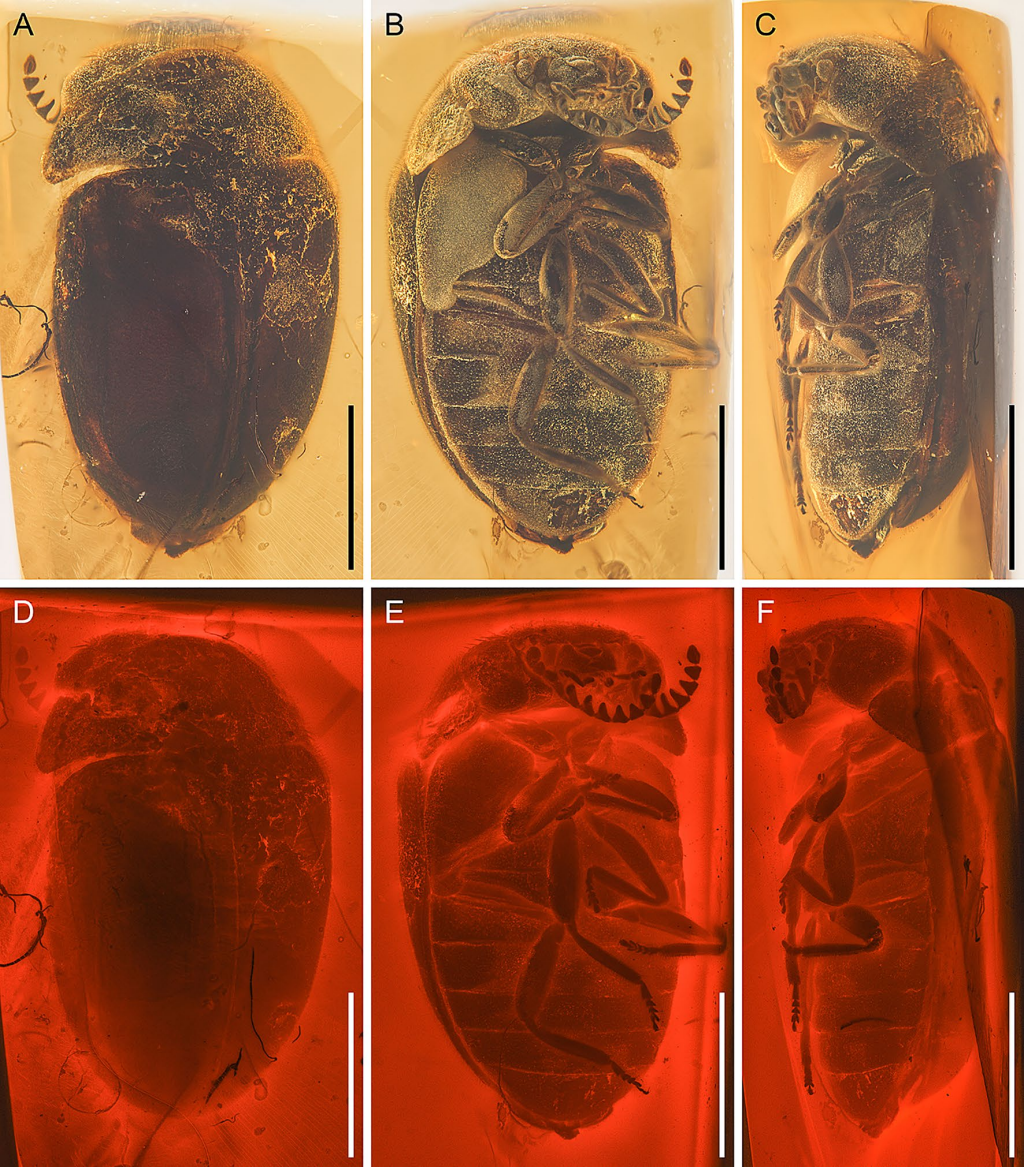
General habitus of *Varcalium lawrencei* **gen. et sp. nov.**, holotype, NIGP177336, under incident light (**A**–**C**) or widefield fluorescence (**D**–**F**). (**A**, **D**) Dorsal view. (**B**, **E**) Ventral view. (**C**, **F**) Lateral view. Scale bars: 1000 μm.

**Figure 2:**
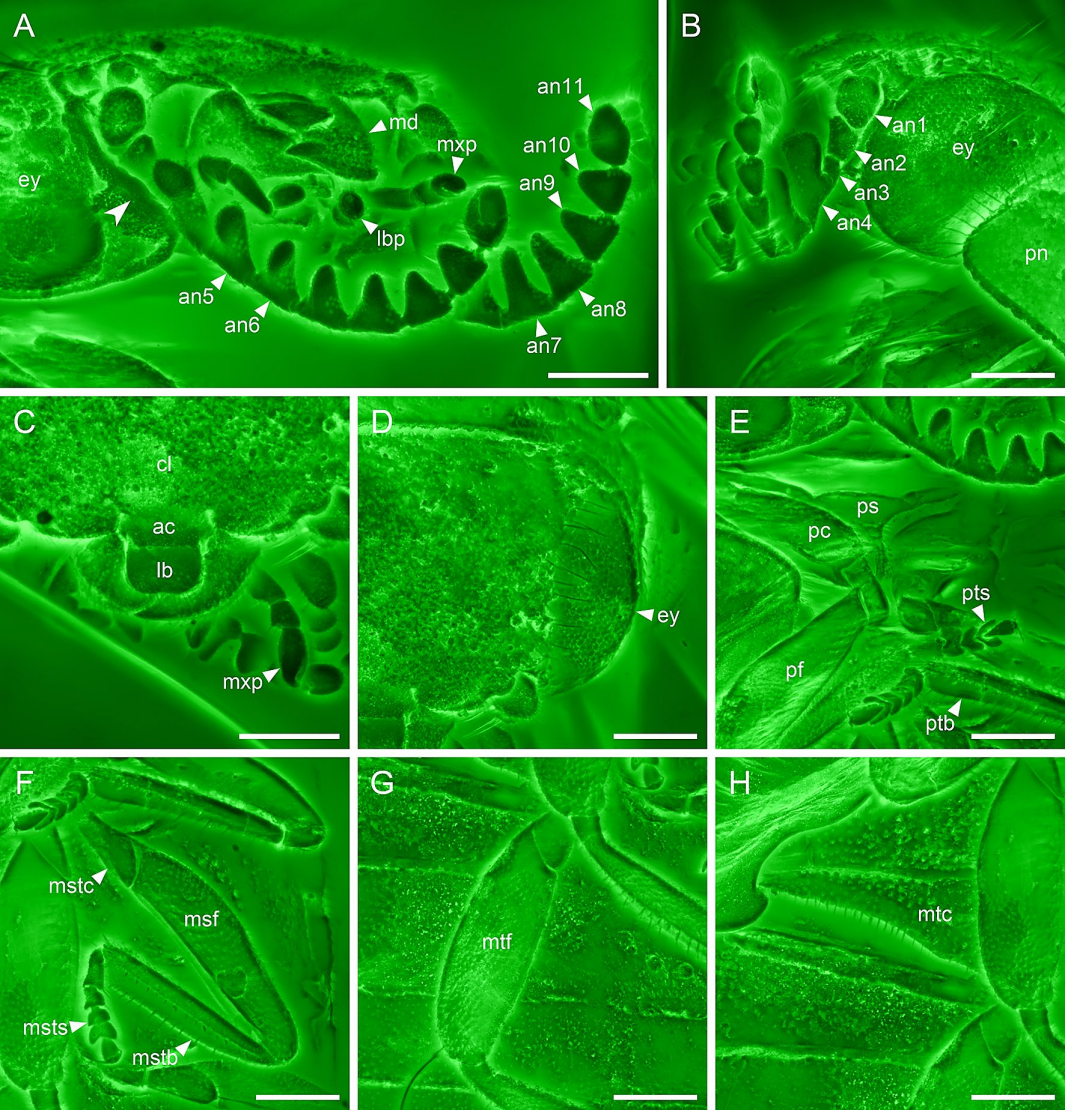
Details of *Varcalium lawrencei* **gen. et sp. nov.**, holotype, NIGP177336, under confocal microscopy. (**A**) Head, anterolateral view. (**B**) Head, lateral view. (**C**) Mouthparts, dorsal view. (**D**) Head, dorsal view. (**E**) Prothorax, ventral view. (**F**) Mid legs. (**G**) Metafemur. (H) Metacoxa. Abbreviations: ac, anteclypeus; an1–11, antennomeres 1–11; cl, clypeus; ey, compound eye; lb, labrum; lbp, labial palp; md, mandible; msf, mesofemur; mstb, mesotibia; mstc, mesotrochanter; msts, mesotarsus; mtc, metacoxal; mtf, metafemur; mxp, maxillary palp; pc, procoxa; pf, profemur; pn, pronotum; ps, prosternum; ptb, protibia; pts, protarsus. Scale bars: 200 μm.

**Figure 3:**
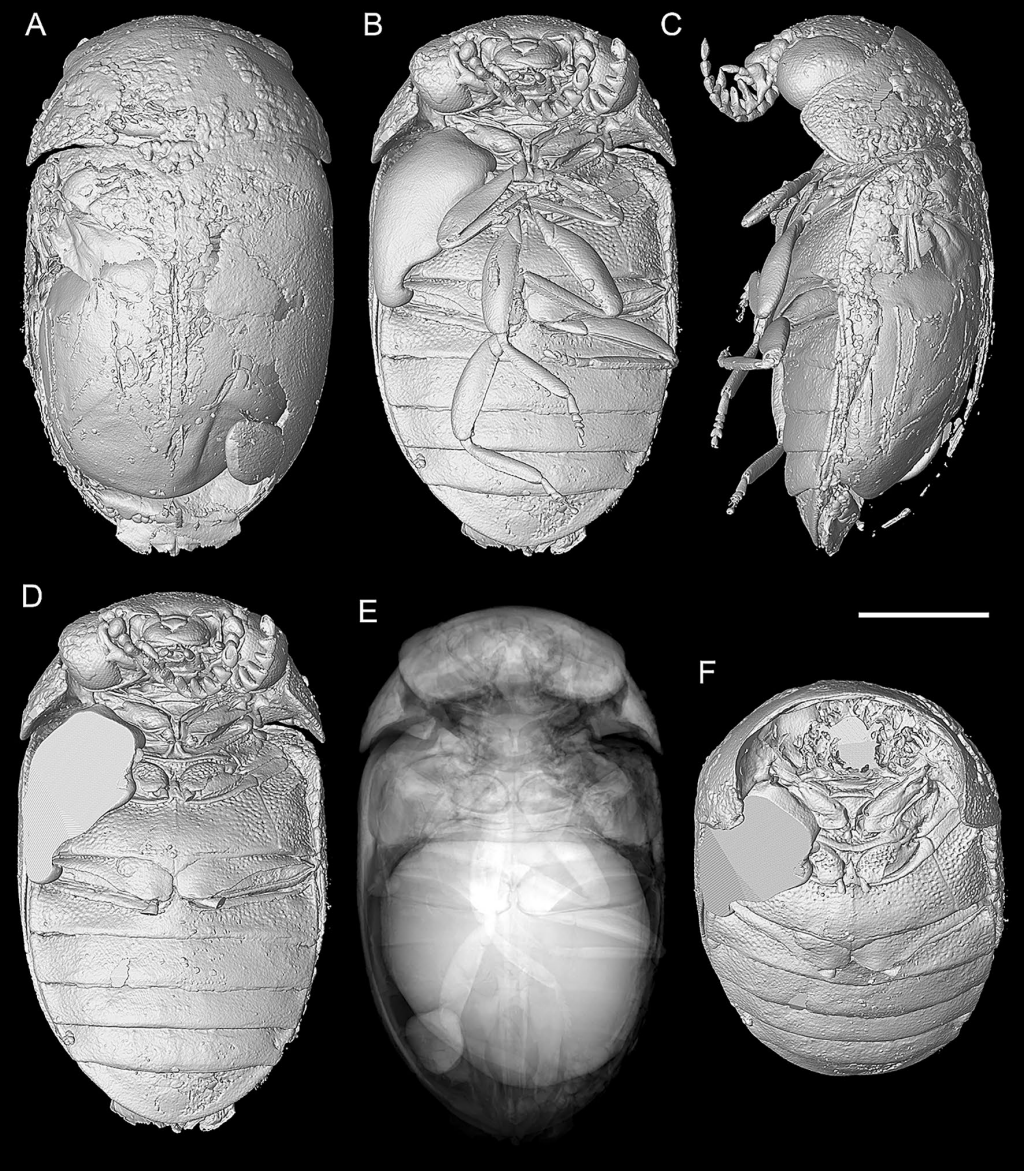
X-ray microtomographic reconstruction of *Varcalium lawrencei* **gen. et sp. nov.**, holotype, NIGP177336. (**A**) Dorsal view. (**B**) Ventral view. (**C**) Lateral view. (**D**) Ventral view, with legs removed. (**E**) Ventral view, rendered under “Sum along Ray” mode. (**F**) Anteroventral view, with head removed. Scale bar: 1000 μm.

**Etymology.** The specific name is a patronym in honour of Dr. John F. Lawrence, an eminent expert in systematics of Coleoptera.

**Type material.** Holotype, NIGP177336 (field number: HUANG-HP-B-3245), male (NIGP).

**Locality and horizon.** Amber mine located near Noije Bum Village, Tanai Township, Myitkyina District, Kachin State, Myanmar; unnamed horizon, mid-Cretaceous, Upper Albian to Lower Cenomanian^45,46^.

**Diagnosis.** As for the genus.

**Description. Adult male.** Body compact, elongate oval, 3.0 mm long, 1.8 mm wide, with irregular punctation and short setae.

**Figure 4:**
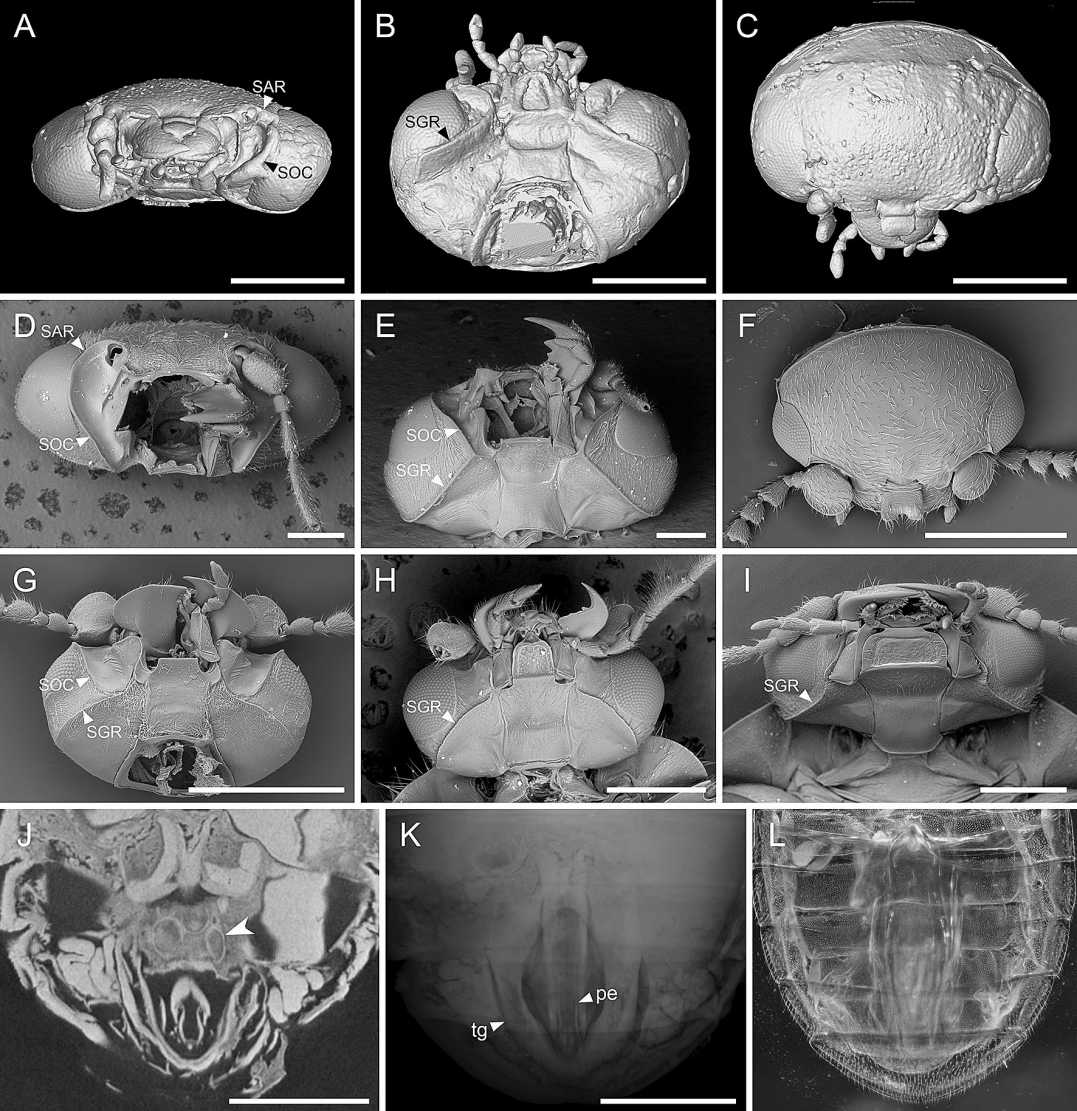
Comparison between *Varcalium lawrencei* **gen. et sp. nov.** and its extant relatives. (**A**–**C**) *Varcalium lawrencei* **gen. et sp. nov.**, head, mirrored, with antennae (partly) removed. (**D**, **E**) *Byrrhopsis* sp., head, with antennae and mouthparts (partly) removed. (**F**, **G**) *Perplexacara latusmandibulara*, head. (**H**) *Prionocyphon ornatus*, head. (**I**) *Macrohelodes* sp., head. (**J**, **K**) *Varcalium lawrencei* **gen. et sp. nov.**, aedeagus inside the abdomen, horizontal section (J) or “Sum along Ray” rendering (K), with arrowhead showing the cuticular rings (**J**). (**L**) *Perplexacara latusmandibulara*, aedeagus inside the abdomen. Abbreviations: SAR, supraantennal ridge; SGR, subgenal ridg; SOC, subocular carina; pe, penis; tg, tegmen. Scale bars: 500 μm.

Head (Fig. 4A–C) about 0.70 times as long as maximum width, with very short tempora, without neck. Distance across eyes about 1.60 times as great as distance between them. Posterior edge of head above occipital foramen with low transverse ridge; upper edge of occipital foramen broadly biemarginate. Eyes (Fig. 2B, D) large; each about as long as half of head width behind eyes, entire and finely facetted, without interfacetal setae. Antennal insertions exposed, widely separated. Antennae (Fig. 2A, Supplementary Fig. S1A, B) about as long as maximum head width, 11-segmented; scape moderately large and broadly ovate, subcylindrical, without sharp ridge on anterior portion; pedicel about half as long and much narrower, subconical, wider at base than at apex; antennomere 3 shorter than 2 and narrow, as wide as long; antennomeres 4–10 uniramose, with the ramus on antennomere 4 thick and club-like; those on the rami of antennomeres 5–7 elongate and flattened (pectinate) and the rami of antennomeres 8–10 gradually shorter and more or less triangular (serrate); antennomeres 4–10 covered with oval sensillae; terminal antennomere elongate and irregularly fusiform. Clypeus transverse, ca. 4.5 times wider than long, deeply emarginate in central portion of anterior margin (Figs. 2C, 4C); frontoclypeal suture absent. Small, transversely rectangular anteclypeus present between clypeus and labrum (Figs. 2C, 4C). Labrum (Figs. 2C, 4C) as long as clypeus and 0.55 times as long as wide, slightly covering mesal portions of mandibles, with straight sides, truncate apex and rounded anterior angles. Mandibles strongly curved, unidentate and overlapping apically (Figs. 2A, C, 4A). Maxilla (Fig. 4B) with relatively small cardo, narrowly elongate stipes and small apical lobe. Maxillary palp with basal palpomere small and globular; second palpomere 2.5 times as long, with oblique apex; third slightly shorter but similar in form and fourth about twice as long as wide, fusiform, widest in the middle of its length and narrowly rounded at apex. Prementum (Fig. 4B) with broadly rounded ligula; labial palpomere 1 long and narrow; palpomere 2 as long as first, much wider and globular; palpomere 3 more than twice as long as second, widest near base and narrowly rounded at apex, arising from apex of preapical palpomere. Mentum (Fig. 4B) subtrapezoidal, about as long as wide, widest posteriorly and gradually narrowing anteriorly, lined with a relatively thick ridge enclosing a flattened depression. Submentum (Fig. 4B) half as long as wide, with curved sides, separated from gula. Gula 0.45 times as long as wide, widest posteriorly and slightly narrowed anteriorly. Subgenal ridges strongly developed, without buttonhole, connected with subocular carinae separating subantennal grooves from eyes (Fig. 4A, B). Subantennal grooves narrowed to maxillary bases (Fig. 4A, B).

Pronotum about 0.42 times as long as wide, considerably wider than head, widest posteriorly; anterior edge more or less truncate; anterior angles more or less right, not produced forward; sides weakly curved with fine marginal carina; disc moderately convex. Scutellar shield subtriangular. Elytra about 1.09 times as long as combined width and 2.64 times as long as pronotum; sides very slightly curved and apices broadly, conjointly rounded; epipleura widest anteriorly and gradually narrowing posteriorly.

Prosternum (Figs. 2E, 3D, F) subtriangular, very short in front of coxae, with very narrow lateral portions; surface flat but with relatively narrow, declined anterior head rest; prosternal process slender, extending to edges of coxae and narrowly rounded at apex. Procoxae large and oblique, with slender plates. Protrochantin exposed and forming narrow strip. Mesoventrite (Fig. 3D) strongly transverse, lateral portions about one-fourth as long as mesocoxae, which are separated by about a third the coxal length; mesoventral process broad at base, narrowing apically and cleft at apex, touching anterior metaventral process. Mesanepisternum short, about 0.5 times as long as wide, shorter in mesal portion. Mesocoxae transversely oval, with distinct plates, broader mesally. Metaventrite (Fig. 3D) about 0.36 times as long (excluding anterior process) as wide; widest posteriorly and slightly narrower anteriorly, with discrimen and with metakatepisternal suture extending from midline about half way to lateral edges. Metacoxae (Fig. 2H) strongly transverse, reaching elytral epipleura, with plates well developed mesally. Metanepisternum broad, about 2.3 times as long as wide, widest at anterior end.

Femora moderately thickened at middle, with tibial groove on inner edge (Fig. 2F, G). Tibiae (Fig. 2F) slender and subequal in length to femora, not expanded apically, with paired longitudinal carinae and distinct apical spurs. Tarsi 5–5–5 (Fig. 2E, F); tarsomere 1 about twice as long as 2; tarsomeres 2–3 relatively wide, subtriangular; tarsomere 4 bilobed; pretarsal claws simple.

Abdomen (Fig. 3D) about 0.80 times as long as wide; ventrite 1 slightly shorter than 2, with acute intercoxal process; ventrites 1–3 apparently connate; 5 broadly rounded at apex. Terminal segments and aedeagus symmetrical (Fig. 4J, K). Penis tubular, with internal structures visible and paired hooked processes at apex. Tegmen well developed, with wide parameres.

## Discussion

### Systematic position of *Varcalium* and comparison with extant relatives

*Varcalium* shares a typical habitus with extant Scirtidae and can be confidently placed within the family based on the combination of the following characters: head with paired subgenal ridges, protrochantins exposed, mesocoxal cavities laterally open, tarsi 5–5–5, penultimate tarsomere lobed beneath. *Varcalium* is similar to the sister group of Scirtidae, Decliniidae, in having serrate antennae (Fig. 2A, Supplementary Fig. S1A, B). However, it clearly differs from Decliniidae in having a marginally bordered mentum, very short prosternum in front of procoxae, and a narrow prosternal process^47^. Besides, a few extant members of Scirtidae also have serrate antennae (e.g., *Prionocyphon* Redtenbacher^19^, *Macrodascillus* Carter^20^, *Prionoscirtes* Champion, and *Mescirtes* Motschulsky^12^).

Scirtidae is currently divided into three subfamilies, Nipponocyphoninae, Stenocyphoninae, and Scirtinae. Nipponocyphoninae and Stenocyphoninae are basal lineages in the family, and both contain a single genus. *Varcalium* clearly does not belong to Nipponocyphoninae or Stenocyphoninae based on the absence of a frontoclypeal suture, unidentate mandibles, and tibiae with paired longitudinal carinae (Fig. 2C, F). Within Scirtinae, several genera (*Exochomoscirtes* Pic, *Ora* Clark, and *Scirtes* Illiger) with very thick hind femora for jumping form a distinct and well-supported clade^29^. *Varcalium* is excluded from this group by the lack of thickened hind femora (Fig. 2F).

**Figure 5:**
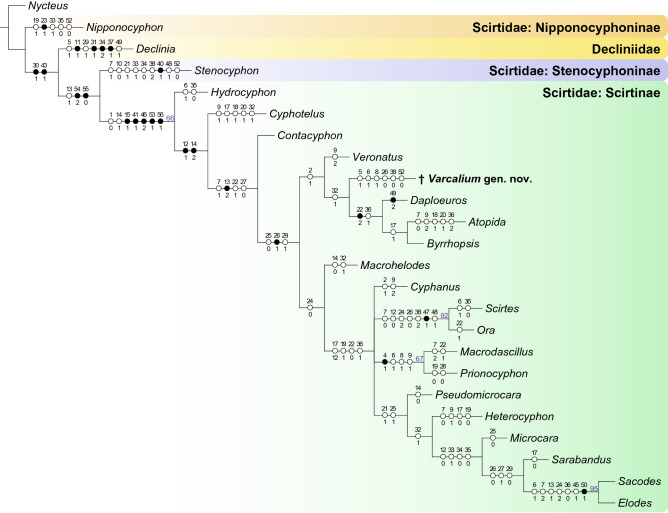
Placement of *Varcalium* **gen. nov.** within Scirtidae. Black circles indicate nonhomoplasious changes; white circles indicate homoplasious characters. Bootstrap support values lower than 60% are not shown.

Ridges on head are important characters in scirtid taxonomy (Fig. 4). In the majority of scirtid genera, the supraantennal ridge (SAR) is short, and ends where it meets the medial edge of the eye, and therefore the supraantennal ridge and subgenal ridge (SGR) are not connected (e.g., Fig. 4H, I^48^). In some other scirtids (e.g., *Atopida* White, *Byrrhopsis* Champion, *Calvarium*, *Mucronotus* Ruta, *Pachycyphon* Zwick^49–52^), the supraantennal ridge turns ventrally in front of eye and becomes the subocular carina (SOC). This subocular carina usually connects the supraantennal ridge with the subgenal ridge, and acts as the outer edge of the subantennal groove (sulcus) in some genera (Fig. 4D–G). In *Varcalium*, the supraantennal ridge smoothly turns ventrally to become the subocular carina, and then again smoothly turns outwards to become the subgenal ridge (Fig. 4A–C). Thus, *Varcalium* is likely related to those extant genera possessing subocular carina. The phylogenetic analysis also supports a close relationship for the genera with subocular carina (Fig. 5). All genera coded in the analysis with subocular carina, except for *Cyphanus* Sharp, form a monophyletic group (*Veronatus* Sharp, *Varcalium*, *Daploeuros* Watts, *Atopida*, and *Byrrhopsis*), although only weakly supported. *Varcalium* is unique in this group in having the subgenal ridge very close to the compound eye (Fig. 4B), while in other members the space between the subgenal ridge and the compound eye is relative wide (Fig. 4E, G) (though in some *Scirtes* the subgenal ridge could be close to the eye^24,53^).

The anterior clypeal margin of *Varcalium* is deeply emarginate in the central portion (Figs. 2C, 4C), which is rarely known in extant Scirtidae, where the anterior clypeal margin is usually straight. A similar deeply emarginate clypeus is known in an unusual member of *Perplexacara* Watts et al., i.e., *P. latusmandibulara* (Watts) (Fig. 4F)^54^. However, it differs from *Varcalium* in scape distinctly enlarged and flattened, forming sharp anterior ridge, and subocular carina weakened posteriorly and not connected with subgenal ridge. *Varcalium* also differs from other extant Scirtidae in the position of mesoventral cavity. In extant Scirtidae (and also Decliniidae), if present, the mesoventral cavity would usually extend into the exposed ventral surface of mesoventrite, leaving a v-shaped notch on the anterior margin of mesoventrite (the line between mesoventrite body and procoxal rests). In contrast, the mesoventral cavity of *Varcalium* is only visible on the surface where procoxal rests develop, and therefore the anterior margin of mesoventrite appears to be intact (Fig. 3D).

Some structures observed in the studied specimen are poorly known in extant Scirtoidea. Very little is known about antennal sensillae (Fig. 2A, Supplementary Fig. S1A,B). Sensillae are present in Eucinetidae^55^, and relatively large sensilla coeloconica are present on antennomeres 6–11 in *Declinia* Nikitsky et al.^47^ (Supplementary Fig. S1D). In Scirtidae sensillar structures were not studied, but are present, especially in genera where modifications of antennae occur, e.g., in *Macrodascillus* (Supplementary Fig. S1C) and *Prionocyphon* (Ruta, unpublished).

Cuticular rings (rectal rings; Fig. 4J) present in an oval sac formed by rectum were reported by Lawrence et al.^47^ in Eucinetidae, Decliniidae, and *Nipponocyphon* (Scirtidae). Similar structures have been recently reported in *Mucronotus velutinus* (Solier) although the putative homology of the structures needs to be confirmed^52^.

### Unexposed beetle aedeagus in amber recovered with micro-CT

Amber fossils offer a potential for three-dimensional preservation of internal structures. The most simple and straightforward way to examine the internal structures of an amber inclusion is to cut open the amber piece. Then the exposed internal structures could be observed with electron microscopy or other methods^56–59^. Alternatively, the amber may be dissolved using chloroform as the solvent, to aid exposing internal structures^60,61^. However, these methods would irreversibly damage the valuable amber specimens. The development of micro-computed tomography (micro-CT) provides an opportunity to investigate the interior of fossils non-destructively. With the aid of micro-CT, researchers have successfully studied the muscles, brain, and even ingested pollen masses within insects entombed in amber^62–66^.

The morphology of male genitalia (aedeagus) is of great importance for the classification of many beetle clades. This is true especially in Scirtidae, a group with a remarkable diversity of genital structures and a generally uniform external morphology within individual genera. However, in beetles fossilised in amber, genitalia are mostly retracted in the abdomen and not externally visible. Micro-CT has been successfully deployed to reconstruct the aedeagi of several beetle fossils preserved in Baltic amber^62,67–77^. By contrast, the unexposed beetle genitalia recovered by micro-CT have been rarely known from Kachin amber, with only one example reported to date^65^. The structure of the aedeagus is highly variable within Scirtidae. Thus, the reconstruction of the aedeagus in the fossil *Varcalium* nevertheless provides some important information on this crucial but still relatively poorly understood character system. Two fundamental studies on the scritid aedeagus and copulation were published by Nyholm^78,79^, and more recently Zwick has revised Nyholm’s hypotheses^80^. While terminal segments are difficult to identify in the studied specimen, it seems that the aedeagus consists of a tubular penis resembling the one of the extant genus *Elodes* while the tegmen is typical for numerous extant genera, like *Microcara* (Fig. 4K).

## Materials and methods

### Material

The Kachin amber specimen studied here was derived from amber mines near Noije Bum (26°20' N, 96°36′ E), Hukawng Valley, Kachin State, northern Myanmar. Jewellery-grade Kachin amber specimens are commonly carried and sold legally in Ruili, Dehong Prefecture on the border between China and Myanmar. The specimen in this study (NIGP177336) was purchased by C.-Y.C. and D.-Y.H in late 2016 from a Myanmar amber dealer (field number: HUANG-HP-B-3245), and is permanently deposited in the Nanjing Institute of Geology and Palaeontology (NIGP), Chinese Academy of Sciences, Nanjing, China. The amber piece was trimmed with a small table saw, ground with emery papers of different grit sizes, and finally polished with polishing powder.

### Imaging

Photographs under incident light were mainly taken with a Zeiss Discovery V20 stereo microscope. Widefield fluorescence images were captured with a Zeiss Axio Imager 2 light microscope combined with a fluorescence imaging system. Confocal images were obtained with a Zeiss LSM710 confocal laser scanning microscope, using the 488 nm Argon laser excitation line. Images under incident light and widefield fluorescence were stacked in Helicon Focus 7.0.2 or Zerene Stacker 1.04. Confocal images were stacked in Helicon Focus 7.0.2. Microtomographic data were obtained with a Zeiss Xradia 520 Versa 3D X-ray microscope at the NIGP micro-CT laboratory and analysed in VGStudio MAX 3.0. Scanning parameters were as follows: isotropic voxel size, 16.916 μm; power, 4 W; acceleration voltage, 50 kV; exposure time, 2 s; projections, 2001. Uncoated specimens of selected species of extant Scirtidae were studied with a S-3400 N Hitachi scanning electron microscope. Images were further processed in Adobe Photoshop CC to adjust brightness and contrast.

### Cladistic analysis

To evaluate the systematic placement of *Varcalium*, we conducted a morphology-based phylogenetic analysis. The data matrix (Supplementary Data S1, S2) was derived from a previously published dataset of Lawrence & Yoshitomi^81^. The genera *Nycteus* Latreille (Eucinetidae) and *Declinia* (Decliniidae, the sister group of Scirtidae) were selected as the outgroup. Since *Macrocyphon* *spencei* Armstrong, which the coding for the genus *Macrocyphon* Pic was based on, has been transferred to *Daploeuros*^20,82^, and *Contacyphon* has been pointed out to be the valid name for *Cyphon* Paykull^83^, the names of these taxa were modified accordingly. The state 2 for character 3 in Lawrence & Yoshitomi^81^ was not present in any of the taxa coded, thus we merged the states 1 and 2. The problematic genus, *Amplectopus* Sharp, which had been placed Chelonariidae^84^, was excluded from the analysis. It shares some superficially similar but likely non-homologous features with Decliniidae^81^, and therefore might interfere the analysis.

Parsimony analysis was performed under implied weights using the program TNT 1.5^85,86^. All characters were treated as non-additive. Parsimony analysis has been shown to achieve the highest accuracy under a moderate weighting scheme (e.g., when concavity constants *K* are between 5 and 20)^87,88^. Therefore, the concavity constant was set to 12 here, as suggested by Goloboff et al.^87^. Most parameters were set as default in the “new technology search”, while the value for “find min. length” was changed from 1 to 500. A strict consensus was calculated. The standard bootstrap analysis was implemented by 10,000 pseudoreplicates, where the support values were shown as absolute frequencies. Character states were mapped onto the tree with WinClada 1.00.08. The tree was graphically edited with Adobe Illustrator CC 2017.

### Nomenclatural acts

This published work and the nomenclatural acts it contains have been registered in ZooBank. The LSID for this publication is url: lsid:zoobank.org:pub:A3E8EF76-2EF3-4081-9817-8352C2A824C0.

## Supplementary Information

Supplementary Information.

## Data Availability

The original confocal and micro-CT data are available from the Zenodo repository (https://doi.org/10.5281/zenodo.6802063).
