# Supplementary material for: A new marsh beetle from mid-Cretaceous amber of northern Myanmar (Coleoptera: Scirtidae)
Source: Sci Rep. 2022 Aug 4;12:13403. doi: 10.1038/s41598-022-16822-y (PMC9352693; doi:10.1038/s41598-022-16822-y)
Supplement: Supplementary file 1 — Supplementary Information. [file 41598_2022_16822_MOESM1_ESM.pdf]

Supplementary Information for

A new marsh beetle from mid-Cretaceous amber of northern Myanmar (Coleoptera: Scirtidae)

Yan-Da Li, Rafał Ruta, Erik Tihelka, Zhen-Hua Liu, Di-Ying Huang, Chen-Yang Cai

List of all Supplementary Information:

Supplementary Figure S1. Antennae of *Varcalium lawrencei* **gen. et sp. nov.** and its extant relatives. (A, B) *Varcalium lawrencei* **gen. et sp. nov.**, with arrowhead showing the sensilla coeloconica. (C) *Macrodascillus scalaris*. (D) *Declinia versicolor*. Abbreviations: an4, 11, antennomeres 4, 11; ey, compound eye. Scale bars: 500 µm in A–C, 100 µm in D.

Supplementary Data S1. List of characters used in the phylogenetic analysis (modified from Lawrence & Yoshitomi, 2007).

Supplementary Data S2. Morphological dataset used for the analysis.

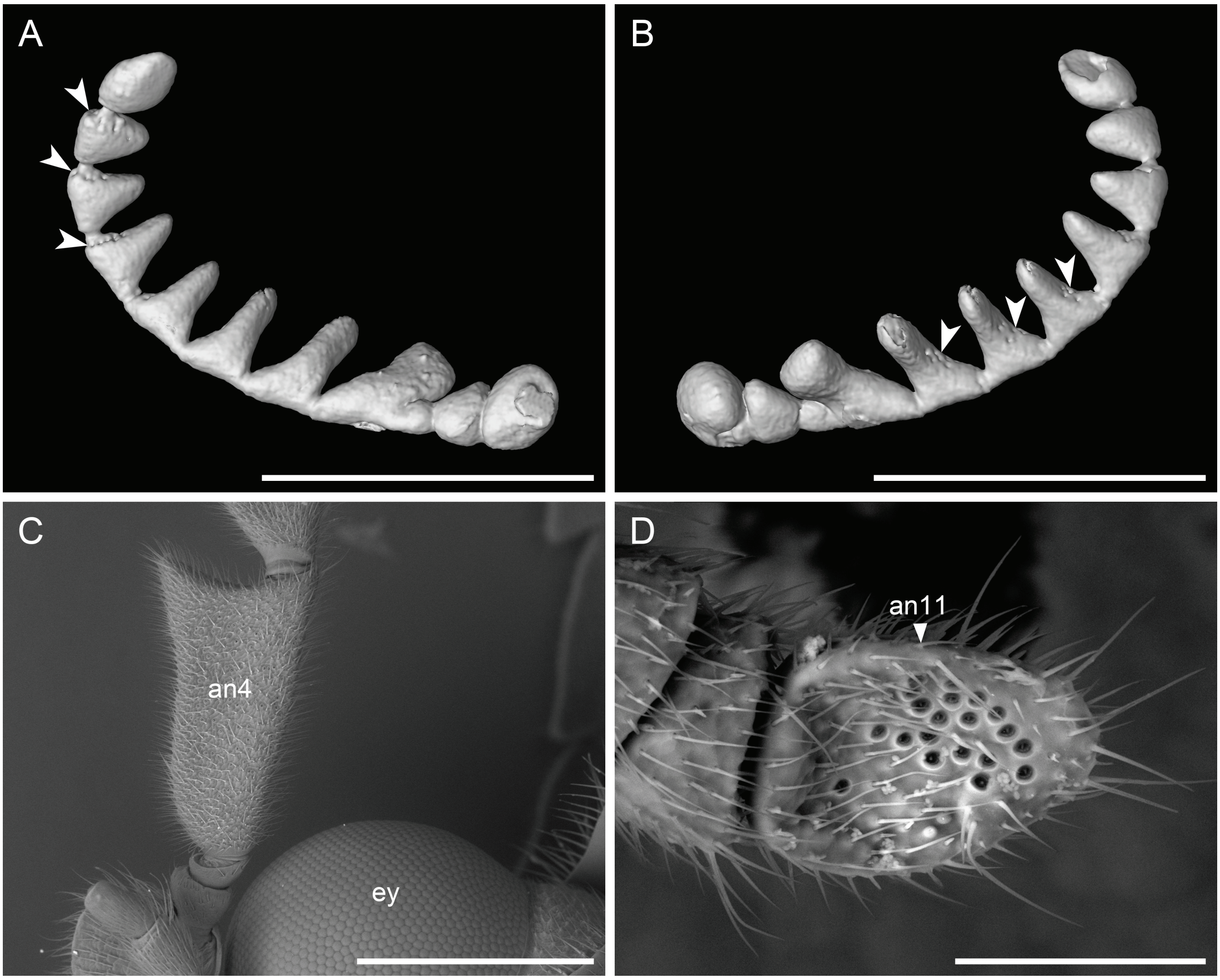

Supplementary Figure S1. Antennae of *Varcalium lawrencei* **gen. et sp. nov.** and its extant relatives. (A, B) *Varcalium lawrencei* **gen. et sp. nov.**, with arrowhead showing the sensilla coeloconica. (C) *Macrodascillus scalaris*. (D) *Declinia versicolor*. Abbreviations: an4, 11, antennomeres 4, 11; ey, compound eye. Scale bars: 500 µm in A–C, 100 µm in D

Supplementary Data S1. List of characters used in the phylogenetic analysis (modified from Lawrence & Yoshitomi, 2007).

1 Frontoclypeal suture: 0, vaguely impressed or absent; 1, distinctly impressed.

2 Subocular carina: 0, absent; 1, present. This refers to a sharp ridge lying between the subgenal ridge and the eye, thus forming one edge of the subantennal groove.

3 Subgenal ridge: 0, absent; 1, present.

4 Antennomere 1: 0, not or only slightly inflated, not carinate; 1, strongly inflated and more or less carinate.

5 Antennomere 2: 0, not wider at base than at apex; 1, distinctly wider at base than at apex.

6 Antennomere 3: 0, not distinctly shorter than 2; 1, distinctly shorter than 2.

7 Antennomere 4: 0, shorter than 2 and 3 combined; 1, between 1 and 2 times as long as 2 and 3 combined; 2, more than 2 times as long as 2 and 3 combined.

8 Antennomeres 4 to 10: 0, neither serrate nor pectinate; 1, serrate or pectinate.

9 Apex of labrum: 0, subtruncate to slightly convex; 1, slightly concave or emarginate; 2, deeply emarginate or bilobed.

10 Mandible: 0, bidentate; 1, unidentate.

11 Dorsal surface of mandible: 0, without carina fitting over sides of labrum; 1, with carina fitting over sides of labrum.

12 Mesal edge of mandible: 0, without teeth or retinacula; 1, with two or more teeth or retinacula.

13 Mesal edge of mandible: 0, with membranous prostheca (sometimes accompanied by fringe of hairs); 1, with fringe of hairs only; 2, with neither prostheca nor fringe of hairs.

14 Mandibular mola: 0, well developed (occupying basal fifth or more); 1, very small (occupying basal tenth); 2, absent.

15 Lacinial apex: 0, with uncus; 1, without uncus. This refers to a sclerotized, hook-like process, usually tridentate.

16 Apical maxillary palpomere: 0, cylindrical to fusiform, not apically expanded; 1, apically expanded and subtriangular.

17 Preapical labial palpomere: 0, not distinctly enlarged or oblique at apex, apical palpomere arising from about middle of apical edge; 1, distinctly enlarged and oblique at apex, apical palpomere arising near inner portion of apical edge; 2, highly distorted, so that apical palpomere arises at middle or near base and palpi appears bifurcate.

18 Prothorax: 0, not widest anteriorly; 1, widest anteriorly.

19 Sides of prothoracic disc: 0, not or slightly explanate; 1, distinctly explanate.

20 Base of prothorax:0, not or slightly narrower than elytral bases; 1, distinctly narrower than elytral bases.

21 Anterior edge of pronotum: 0, truncate or emarginate, not forming continuous curve with lateral edges; 1, strongly rounded, forming continuous curve with lateral edges.

22 Anterior angles of pronotum: 0, absent or not produced forward; 1, produced and rounded or broadly angulate; 2, produced and acute.

23 Lateral pronotal carinae: 0, simple or minutely crenulate; 1, denticulate.

24 Posterior angles of pronotum: 0, absent or broadly rounded; 1, obtuse or right; 2, moderately to strongly acute.

25 Pronotal disc just in front of posterior edge: 0, without pair of small pits; 1, with a pair of small pits.

26 Prosternal process: 0, not abruptly bent, broadened and flattened at apex; 1, abruptly bent, broadened and flattened at apex.

27 Prosternal process ventrally: 0, extending almost to coxal apex; 1, ending well before coxal apex.

28 Protrochantin: 0, large and subquadrate, lying between coxa and edge of notum, forming part of lateral thoracic wall; 1, small and narrow, lying in front of coxa and not forming part of thoracic wall.

29 Anterior edge of scutellum: 0, not or gradually elevate; 1, abruptly elevated forming sharp ridge.

30 Elytral punctation: 0, distinctly seriate; 1, not distinctly seriate.

31 Elytral epipleuron: 0, narrowed apically; 1, slightly widened at apex.

32 Mesoventrite: 0,divided by longitudinal groove or discrimen; 1, not divided by longitudinal groove or discrimen.

33 Mesoventral cavity: 0,present; 1, absent. This refers to a distinct depression lying between and in front of the mesocoxal cavities and not just as light widening of the mesothoracic discrimen.

34 Mesocoxal cavities: 0, contiguous; 1, narrowly separated; 2, moderately to widely separated (more than 0.4× shortest diameter of coxal cavity).

35 Apex of mesoventral process: 0, not cleft or emarginate; 1, cleft or emarginate.

36 Metathoracic discrimen: 0, complete to base of intercoxal process; 1, incomplete but more than half median length of ventrite (excluding intercoxal process); 2, less than half median length of ventrite (excluding intercoxal process).

37 Metaventrite, metepisternum and anterior portion of epipleuron: 0, without impressions for housing mid legs; 1, with impressions for housing mid legs.

38 Metacoxal plate: 0, extending to lateral edge of coxa; 1 ,extending beyond middle of coxa but not to lateral edge; 2, not extending to middle of coxa.

39 Metendosternite: 0, without ventrolateral processes; 1, with ventrolateral processes.

40 Radial cell of hindwing: 0, forming equilateral triangle; 1, forming elongate triangle.

41 Medial field of hindwing: 0, with 4 or more terminal veins; 1, with 3 or fewer terminal veins.

42 Cross-vein joining MP1+2 and MP3+4: 0, present; 1, absent.

43 Wedge cell of hind wing: 0, present; 1, absent.

44 Wing vein AA4: 0, not meeting to anal fold; 1, meeting anal fold.

45 Wing vein AP3+4: 0, forked to form AP3 and AP4; 1, simple or absent.

46 Mesotibia: 0, without longitudinal carina; 1, with single longitudinal carina; 2, with paired longitudinal carinae.

47 Metafemur: 0, not much wider than mesofemur; 1, much wider than mesofemur.

48 Metatibial spurs: 0, more or less equal in length; 1, greatly differing in length.

49 Number of basal ventrites connate: 0, none or two; 1, three; 2, four.

50 Anterior edge of ventrite1 (sternite III): 0, with intercoxal process; 1, without intercoxal process.

51 Spiracles on segment VIII: 0, present; 1, absent.

52 Base of sternite VIII in male: 0,with parallel or diverging lateral struts; 1,with lateral struts meeting to form closed basal rim.

53 Base of segment IX in male: 0, closed forming genital ring; 1, open with separate basal struts.

54 Proctiger (tergite X) in male: 0, completely free from tergite IX; 1, partly fused to tergite IX; 2. completely fused to tergite IX.

55 Rectal rings: 0, absent; 1, present. These structures are illustrated in Lawrence et al. (1995: fig. 17).

56 Parameres: 0, basally articulated; 1, fixed or absent.

Supplementary Data S2. Morphological dataset used for the analysis.

Nycteus	0110010010000000000000100001100000112000000000100100010010
Declinia	1010100001100001000000011000111002111010001111001011001 0
Nipponocyphon	1010000001000001001000111001000011000110010110000010011 0
Stenocyphon	1010001000001000000001001101001001010021101111001001002 00
Cyphotelus	001000001101121011010001101101010110011011111200001112 01
Atopida	011000002101221011010201010111010112011011111200001112 01
Veronatus	011000102101221000000101010111000110011011111200001112 01
Macrohelodes	001000100101201000000100010111010110011011110200001112 01
Macrodascillus	001101211101221010100100010111000111011011110200001112 01
Daploeuros	011000101101221000000201010111010111011011111200201112 01
Pseudomicrocara	001000100101201010101000110111000111011011110200001112 01
Byrrhopsis	011000101101221010000201010111010111011011111200001112 01
Heterocyphon	001000001101211000001001110111010111011011110200001112 01
Cyphanus	011000102101221020100000010111000111011011111200001112 01
Prionocyphon	001101111101221020000000000111000111011011110200001112 01
Sarabandus	001000100100221000101001101101011001011011110200001112 01
Microcara	001000100100221020101000010111011001011011110200001112 01
Sacodes	001001200100121020101002101101011000011011111200011112 01
Elodes	001001200100121020101002101101011000011011111200011112 01
Scirtes	001001000100221020100002000111000110021011111211001112 01
Ora	001000000100221020100102000111000111021011111211001112 01
Hydrocyphon	001001000100111000000001101101000101011011111200001112 01
Contacyphon	001000100101221000000101100101000110011011111200001112 01
Varcalium **gen. nov.**	01101111010????000000101?001?101011000???????20010?01??1
